# Key stakeholders’ views on the quality of care and services available to frail seniors in Canada

**DOI:** 10.1186/s12877-018-0969-y

**Published:** 2018-11-26

**Authors:** Anik M. C. Giguere, Elina Farmanova, Jayna M. Holroyd-Leduc, Sharon E. Straus, Robin Urquhart, Valerie Carnovale, Erik Breton, Selynne Guo, Nandini Maharaj, Pierre J. Durand, France Légaré, Alexis F. Turgeon, Michèle Aubin

**Affiliations:** 1 Department of Family Medicine and Emergency Medicine, Laval University, Pavillon Ferdinand-Vandry, room 2881-C, 1050 avenue de la Médecine, Quebec, QC G1V 0A6 Canada; 2 Quebec Centre for Excellence in Aging, St. Sacrement Hospital, Quebec, QC Canada; 3 Laval University Research Centre on Primary healthcare and services, Quebec, QC Canada; 4 Laval University Research Centre of the CHU de Quebec, Population Health and Optimal Health Practices Unit, Quebec, QC Canada; 5 Section of Geriatric Medicine, Departments of Medicine and CHS, University of Calgary, Calgary, AB Canada; 6 Department of Medicine, University of Toronto, Toronto, ON Canada; 7 Department of Surgery, Dalhousie University, Halifax, NS Canada; 8 School of Population and Public Health, University of British Columbia, Vancouver, BC Canada; 9 Department of Social and Preventive Medicine, Laval University, Quebec, QC Canada; 10 Department of Anesthesiology and Intensive Care, Division of Critical Care Medicine Laval University, Quebec, QC Canada

**Keywords:** Delivery of health care, Frailty, Health planning, Health services needs and demand, Quality of health care, Quality improvement

## Abstract

**Background:**

Frail seniors often receive ineffective care, which does not meet their needs. It is still unclear how healthcare systems should be redesigned to be more sensitive to the needs and values of frail seniors and their caregivers. We thus aimed to describe key stakeholders’ perspectives on the current healthcare and services available to frail seniors.

**Methods:**

In this qualitative descriptive study, we conducted semi-structured interviews with a convenience sample of 42 frail seniors, caregivers, clinicians, or healthcare administrators/decision makers involved in frail senior care from five Canadian provinces. We explored participants’ perspectives on the quality of care and services for frail seniors. We used an inductive/deductive thematic data analysis approach based on the Square-of-Care model, including emerging themes using the constant comparison method.

**Results:**

We grouped participants’ perspectives into strengths, weaknesses and opportunities for improvement, and then into nine themes: care processes, continuity of care, social frailty, access to healthcare and services, models of healthcare delivery, cost of care, healthcare staff management and professional development of healthcare providers, material resources and environmental design of healthcare facilities, and coordination of care. Our findings suggest redesigning assessment, communication with frail seniors and their caregivers, targeting care and services to the needs, and integrating care better across settings and in time.

**Conclusions:**

A systematic identification of frail older people is the first step to adapt healthcare systems to this population’s needs. Participation of frail older people and their caregivers to decision making would also allow choosing care plans meeting their care goals. The integration of care and services across settings, over time, and with various providers, is also needed to meet frail senior needs.

**Electronic supplementary material:**

The online version of this article (10.1186/s12877-018-0969-y) contains supplementary material, which is available to authorized users.

## Introduction

Frailty is a clinically recognizable state of vulnerability caused by aging-associated decline across multiple physiologic systems, which compromise the ability to cope with normal or minor stresses [1]. Compared to their age-matched non-frail counterparts, frail seniors are at much higher risk of fall, infection, hospitalization, institutionalization, and death [1, 2]. Frailty represents a global health concern due to its multiple clinical and societal consequences and accelerated aging of populations worldwide.

In Canada, an estimated 5 to 15% of people are frail, depending on the province [3]. Frail seniors are high users of health services, which translate into a greater number of visits to healthcare providers, more hospital admissions, longer hospital stay, higher use of home care services, and more visits to the emergency department [4–7].

Frail seniors often receive ineffective and even harmful care [8]. They commonly face care coordination and safety problems due to lack of communication between physicians [9]. Many community-dwelling frail seniors do not receive continuing care by the same provider, which results in (preventable) visits to emergency rooms and medical escalation [10]. Such gaps in care may introduce additional health risks, unnecessary financial and social costs associated with recurrent admissions, loss of independence and diminished quality of life [8].

It is still unclear how healthcare systems should be redesigned to be more sensitive to the needs and values of frail seniors and their caregivers. Therefore, we sought to describe the perspectives of users, healthcare providers and decision makers about the current state of the healthcare system for frail seniors and specific opportunities for improvement.

## Methods

In this qualitative descriptive study, we conducted in-depth interviews with key stakeholders from several Canadian provinces, including Quebec (QC), Nova Scotia (NS), Ontario (ON), British Columbia (BC), and Alberta (AB).

### Participants

We recruited a convenience sample of frail seniors, caregivers, healthcare providers (HCP), and administrators/decision makers from nursing homes, hospices, hospitals, and home care agencies (DM). We recruited DM and HCP through the networks of the research team members. Patients and caregivers were recruited through the participating HCPs or through posters in geriatric clinics where the participating HCP worked. Seniors were eligible to participate if they were 65 years of age or older, and considered frail according to the Clinical Frailty Scale [11] or the Edmonton Frail Scale [12]. Frail seniors with cognitive impairments were also eligible to participate if their caregiver accompanied them, and if their caregiver agreed to participate and answer the interview questions if the frail senior was unable to do so.

### Data collection

Six members of the research team conducted the interviews from June to October 2015, to explore the participants’ views and experiences with healthcare and services for frail seniors, and to solicit their perspectives about potential opportunities for improvements. The 50-min interviews followed a semi-structured guide (Table 1), which we adapted for frail seniors and their caregivers to avoid jargon. We conducted phone interviews with DM and HCP, and in-person interviews with frail seniors and caregivers. The interviews were audio recorded, and transcribed verbatim. One participant refused recording but agreed to note taking as an alternative.

**Table 1:** Interview guide

1. How are you interested/involved in the care of frail seniors?	
2. In your opinion, what, if anything, is different about the provision of care to frail seniors compared to other patients?	
3. In your opinion, what are the most important components of quality of care for frail seniors? *Probe:* – *What components of healthcare services, resource utilization, models of care are especially important for this population?*	
4. Do you know of any instances where frail seniors did not receive quality care? If so, why didn’t they receive quality care? *Probes:* – *Were there particular things about their health problems that might help explain why they did not receive quality care?* – *Were there aspects of their psychological health or social environment that might help explain why they did not receive quality care?* – *Were there aspects of their family environment that might help explain why they did not receive quality care?* – *Were there things about the establishment in which they received care that might help explain why they did not receive quality care?*	
5. Imagine a future ten years from now and that your province was well organized to care adequately for frail seniors. What does that future look like to you? *Probes:* – *What would high quality care and services look like?* – *In contrast, what would poor quality care and services look like?*	

### Data analyses

The thematic data analyses combined deductive and inductive approaches. The Square-of-Care conceptual framework [13] initially guided the deductive analysis. This framework guides palliative care and describes a comprehensive set of care processes (e.g. assessment, information sharing, decision making) and issues associated with illness and bereavement (e.g. social, psychological, physical, practical issues). An inductive analysis allowed including new emerging themes using the constant comparative method of analysis [14]. Three researchers collaborated for the analyses: one initially analysed the data (EB), another validated the findings (VC), and the third verified consistency between the themes and the content of interviews (AMCG). The team met regularly to review codes and resolve discrepancies through discussion. A qualitative data analysis software (NVivo version 10, QSR International) facilitated the analyses.

## Results

We interviewed 42 participants: eight frail seniors and/or their caregivers, 18 HCP, and 16 DM (Tables 2, 3, 4 and 5). In QC and BC, we recruited frail seniors or their caregivers through participating HCPs. In the other provinces where we used posters in geriatric clinics, recruitment of frail seniors or their caregivers remained unsuccessful despite all efforts. Most of the participating HCP were physicians (*n* = 11), and more than half specialized in geriatric care. The other HCP were nurses (*n* = 4), and social workers (*n* = 3). The majority of participating DM worked in provincial health systems. We did not recruit any frail senior with cognitive impairment.

**Table 2:** Socio-demographic characteristics of participants (DM = decision maker, HCP = healthcare professionals)

Characteristic	DM (*n* = 16)	HCP (*n* = 18)	Frail senior (*n* = 5)	Caregiver (*n* = 3)
All participants				
Gender				
Female	13	11	1	2
Male	3	7	4	1
Age (years)				
25–34		1		
35–44	1	5		
45–59	15	6		2
60–64		4		
65+		2		
65–74			1	
75–84			2	
85+			2	
NA				1
Province				
AB	4	4		
BC	4	4	3	1
NS	2	2		
ON	1	4		
QC	5	4	2	2

**Table 3:** Socio-demographic characteristics of decision makers (DM)

Characteristics	Frequency (*n* = 16)
Management experience (years)	
6–10	4
11–15	2
16–20	2
21–25	5
26–30	2
31–35	1
Type of organization	
Provincial health system	10
University	1
Hospital	1
Senior Advocate	1
Medical Association	2
Regional Health Agency	1
Level of organization	
Regional	3
Provincial	11
National	2
Role in the organization	
Operations	5
Planning	1
Operations, planning and finances	2
Other	8
Educational background	
MD	3
MA public administration	2
MD, CCFP	2
RN, MN, BSN	2
MBA	1
M. Sc. Health Services Administration	1
BScN	1
BScPT, MSW, MBA	1
FRCPC int.Medecine	1
BA, MBA,,MSC	1
B.Sc. Health promotion, M.Sc. Health administration, Certified health executive (CHE)	1

**Table 4:** Socio-demographic characteristics of healthcare professionals (HCP)

Characteristics	Frequency (*n* = 18)
Profession	
Physician	11
Nurse	4
Social Worker	3
Specialization in geriatric care	
Yes	11
No	7
Practice experience (years)	
1–5	4
6–10	1
16–20	3
21–25	3
26–30	2
31–35	2
36–40	2
N/A	1
Language used at work	
English	14
French	4

**Table 5:** Socio-demographic characteristics of patients and caregivers

Characteristics	Frequency (*n* = 8)	
	Patient (*n* = 5)	Caregiver (*n* = 3)
Language at home		
English	3	1
French	2	1
Other		1
Patient’s marital status		
Married or domestic partnership	4	2
Single		1
Widow	1	
Patient’s location		
At home	2	
Nursing home		1
Other		
Retirement home	1	2
Extended care unit	1	
At home and in a hospice	1	
Caregiver living with patient		
No		3
Patient have a caregiver		
Yes	3	
No	1	
N/A	1	
Patient’s health problem		
Arthritis	2	
Parkinson		1
Paralysis		1
N/A	3	1

**Fig. 1:**
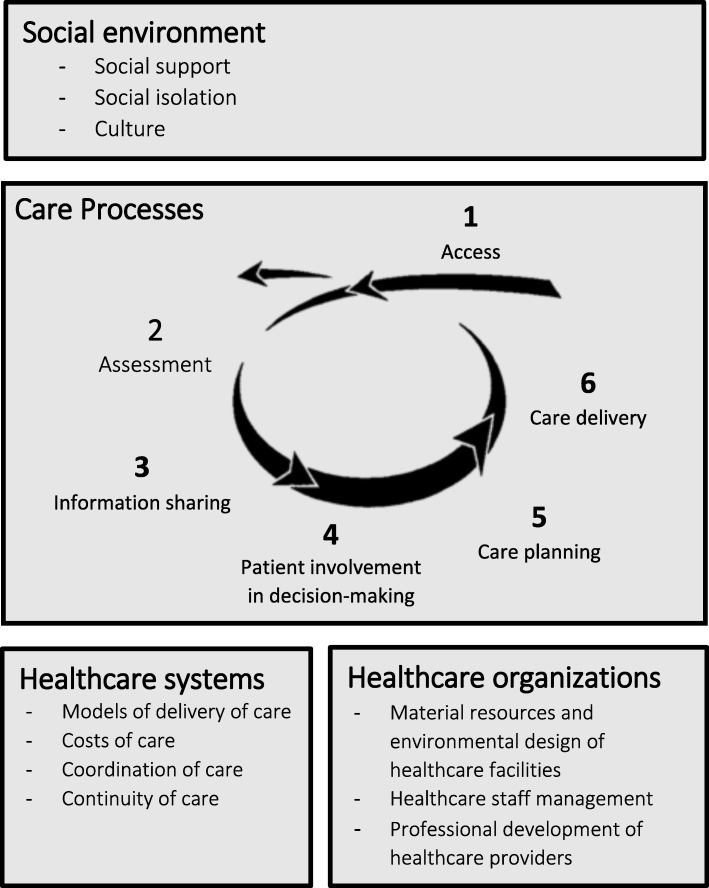
Key features of the quality of care and services for frail seniors, as initially deduced from the Square-of-Care conceptual framework [13], and then induced by study participants

The final qualitative analysis retained several of themes describing care processes in the Square-of-Care conceptual framework (assessment, information sharing, care planning, care delivery) [13]. After our analysis, the Square-of-Care theme ‘Decision-making’ was renamed ‘Patient engagement in decision-making’, and we added a new theme, ‘Access’, to the list of care processes proposed in the framework. We also added several other themes raised by study participants, to propose a set of key features of the quality of care and services available to frail seniors (Fig. 1). We further categorized these themes into weaknesses (Table 6), strengths (Table 7), and opportunities for improvement. A more detailed account of the subthemes is available in the (Additional file 1: Appendices 1–15).

**Table 6:** Frequency of participants who discussed weaknesses of the current healthcare services for frail seniors in Canada, by main theme (and sub-theme), and by Canadian province (AB = Alberta, BC = British Columbia = Nova Scotia, ON = Ontario, QC = Quebec)

	Overall (*n* = 42)	Province				
		AB (*n* = 8)	BC (*n* = 12)	NS (*n* = 4)	ON (*n* = 5)	QC (*n* = 13)
Care processes						
1. Access to healthcare and services	19	3	5	3	3	5
2. Assessment	20	7	5	2	1	5
3. Information sharing	8	2	3	2	0	1
4. Patient engagement in decision-making	9	2	4	0	1	2
5. Care planning	3	1	0	1	0	1
6. Care delivery	29	6	10	4	2	7
Social environment						
Social support	16	5	4	2	3	2
Social isolation	8	2	2	1	2	1
Culture	1	1	0	0	0	0
Healthcare systems						
Models of delivery of care	18	6	4	3	1	4
Cost of care	17	4	3	2	2	6
Continuity of care						
Relational continuity	10	0	2	0	1	7
Informational continuity	6	1	3	0	1	1
Management continuity	1	0	0	0	0	1
Coordination of care	9	1	3	1	3	1
Healthcare organizations						
Healthcare staff management and professional development of HCP	19	5	7	0	3	4
Material resources and environmental design of healthcare facilities	11	4	5	0	1	1

**Table 7:** Frequency of participants who discussed strengths of the current healthcare services for frail seniors in Canada, by main theme (and sub-theme), and by Canadian province (AB = Alberta, BC = British Columbia = Nova Scotia, ON = Ontario, QC = Quebec)

	Overall (*n* = 42)	Province				
		AB (*n* = 8)	BC (*n* = 12)	NS (*n* = 4)	ON (*n* = 5)	QC (*n* = 13)
Care processes						
1. Access to healthcare and services	3	0	2	0	0	1
2. Assessment	3	0	0	0	1	2
3. Information sharing	0	0	0	0	0	0
4. Patient engagement in decision-making	4	0	2	0	1	1
5.Care planning	1	0	0	0	0	1
6. Care delivery	8	1	2	0	1	4
Social environment						
Social support	9	1	3	1	1	3
Social isolation	4	2	1	0	0	1
Healthcare systems						
Models of delivery of care	4	2	1	0	1	0
Cost of Care	1	1	0	0	0	0
Continuity of care						
Relational continuity	2	0	1	0	0	1
Management continuity	2	1	0	0	0	1
Healthcare organizations						
Healthcare staff management and professional development of HCP	6	2	0	1	2	1
Material resources and environmental design of healthcare facilities	2	1	1	0	0	0

### Care processes

#### Access to health care and services

Participants perceived that long wait times for accessing acute care and specialized services, and limited access to primary care, often result in using emergency care. Essential services, which would prevent crises, often remain inaccessible. There is also a mismatch between what frail patients qualify for, and what they truly need:

*“Because my father is over 65, he doesn’t get any rehabilitation. And that kind of shocked us because my dad… one of his main hobbies is walking and we kind of thought that you’d want to focus on rehab to get him some level of capability, so he’s not such a burden on the healthcare system. And we were told no. There’s no money for that. He does not qualify.” (BC, Caregiver#2)*

An HCP explained that qualifying criteria for subsidized assisted living are so narrow that they leave out many patients who would have benefited from it:

*“I think that’s a real gap. Unfortunately, people then... don’t move when they might want to, waiting for a crisis to happen.” (BC, HCP#2)*

Participants also highlighted that levels of care often do not match the changing needs of frail seniors. Frail seniors and their families find themselves in a ‘vicious cycle’, as they are waiting for a certain level of care, which, when they get it, does not match anymore their needs that have changed:

*“…once they are in the nursing home, we see people, you know, fall and break a hip, for example, and then require higher level of care. But the nursing home doesn't always allow for a gradual increase in the level of care. They may have been on the waiting list to get in that nursing home for a lower level of care, and then because of a health event that requires more care, they stay in the hospital. They can lose their spot in the nursing home and have to wait for a new level of care…” (NS, HCP#2)*

The essential access of frail seniors to primary care services is also complicated by the retirement of family doctors, which often leaves frail seniors stranded.

*“A lot of the frail seniors cannot access primary care, Right now, a lot of the older physicians are retiring from their practice and they are not taking on new patients; this is just what I’ve seen. As a result, older people can’t find primary care physicians to take on their case because they are so frail.” (AB, HCP#2)*

#### Assessment

Half the participants described assessments as inadequate, as underlined by this decision maker:

*“There are plenty of people who do not have an answer to their needs because they are not detected quickly. More and more, our partners are sensitized to monitor signs of loss of autonomy. I think that our geriatricians who practice in remote areas are very cooperative in supporting front-line physicians in identifying this loss of independence.” (QC, DM#5)*

Participants also emphasized the importance of comprehensive geriatric assessments to diagnose frailty, and underlined difficulties accessing it. They also judged mental health assessments, and time dedicated to assess patients, as inadequate. Participants stressed the importance of improving investigations of falls, cognitive impairment, and polypharmacy:

*“An individual that I've seen recently presented with falls. The person was seen by a fall's service and was put into a balance training exercise program, but unfortunately, the person had a neurological problem for their fall difficulty, which would have required a search intervention. People assumed that all the person needed was an exercise program, when they actually needed a diagnostic evaluation. A new unrecognized problem was causing the presentation. No one stepped back to see if there might be some something new going on.” (AB, HCP#1)*

#### Information sharing

The sharing of health information among frail patients, their caregivers and HCP was reported as problematic. Participants reported that caregivers lacked access to health information regarding the frail patient. They also reported a lack of health information to facilitate interactions and discussions among patients, caregivers and HCP:

*“The system is configured on the assumption that people can speak for themselves and have all the information, whereas when you’re dealing with seniors, often times you’re dealing with the family. They bring their own perspective; they also bring additional information to the encounters, which isn’t always respected or considered.” (NS, HCP#1)*

Participants also underlined the lack of mechanisms allowing caregivers access to important health information, such as discharge instructions, as explained by this decision maker:

*“And then for many older people, especially with those who have cognitive impairment, there needs to be a lot of family involvement. Sometimes, I think that with all our privacy policies we forget to involve the family. Therefore we may give the individual the information that they would need, but that doesn't mean that they can actually use what you have given them and actually apply it to their lives.” (NS, DM#1)*

#### Patient involvement in decision-making

Participants also reported several weaknesses related to the lack of engagement of patients in decision-making:

*“Because they are frail, [they] may be not treated as equal as citizens… not talked to directly. Their families maybe talked to, or their caregivers, as opposed to them… They are not included in the conversation. […] They are not involved and they want so desperately to be independent, respected and included, of course, in their healthcare decisions.” (BC, HCP#2)*

Participants perceived that the lack of patient engagement in decision-making to be widespread:

*“At this time, frail older adults do not have many decisions to make on their own. Healthcare establishments make every decision for them along their trajectory… Essentially, frail people have no control, none whatsoever… not over their lives and care. […] If we want to improve the quality of care, frail older adults must be empowered to partake in their care.” (QC, DM#4)*

#### Care planning

Participants reported the reluctance of caregivers and family members to engage in advanced care planning. They mentioned the need for well-developed procedures and resources to ensure that caregivers and families are duly and timely informed and educated, to prepare them for decline and engage them in end-of-life care:

*“We need to be better equipped to have these discussions, and to help us, seniors and the caregivers make informed decisions around a plan. […] Our older adults and the family caregivers should be given the right information so they can make informed decisions.” (AB, HCP#4)*

*“I think end-of-life care discussions are an important thing that are missed often. When you’re working in geriatrics, you are certainly facing end-of-life care decisions. I think it’s difficult for a lot of people to have that talk. I think that it’s something that geriatrics does very well. Otherwise, people don’t get to talk about their hopes and plans for their end-of-life goals of care, when to end treatment. If somebody is on chemo dialysis and they’re reaching the point where they just don’t want to go on anymore, and nobody has that conversation with them, then they don’t know that they can say, I’ve had enough. I want to stop now and know what palliative care looks like.”(ON, HCP#4)*

The participants also raised the issue of the lack of adherence to care plans, as underlined by a decision maker from Nova Scotia:

*“Sometimes when people are taken to emergency, even if they have some kind of a care plan that may have been made with their doctor or their geriatrician or some other kind of healthcare provider around the limits of that care, which care plan isn’t always accessible or available at emerge. So the limits of that care plan may not always be respected.”(NS, DM#2)*

Patients and caregivers suggested that adherence might be improved if patients and caregivers were included as formal members of the care team:

*“Sometimes, more players need to be engaged in the planning for healthcare support. If the person has a family care provider who will be assisting, either when they are in hospital or when discharged from hospital, we should engage that person into the team of care.” (AB, DM#4)*

#### Care delivery

Participants also perceived care delivery as lacking sensitivity to frailty:

*“…the person had to wait four days before they actually had the hip surgery. And every one of those days, the person wasn’t allowed to eat just in case they would get the operation that day. So the family and the individual, who was 90 years old, felt that this was an example of a frail elder who was not given high priority in our healthcare system.” (BC, DM#3)*

Some participants expressed concerns about the stigma and prejudices against frailty in healthcare settings, as discussed by this decision maker form Quebec:

*“The other thing that makes frail seniors different, I think, is stigma. I think there is a large stigma by us against older patients. And I find in the healthcare system that, when a senior with a complex medical profile doesn't fit in a box like the hospital box, or the family physician box, or the long-term care box, then it's often the case that nobody wants them and they're... they're truly abandoned by the system.” (QC, DM#1)*

The inappropriate use of medications, the designs of acute care settings, and the management of mental health issues also worried them:

*“So in residential care, one of the biggest problems is the use of antipsychotics to help people sleep or be less agitated, and they get in a cycle of alienation, really.” (BC, HCP#3)*

Participants emphasized the importance of the frail patients’ quality of life:

*“My dad has zero quality of life. He talks regularly that “can you just cremate me?” […] He’s gradually getting a little bit of dementia… because for two years, no mental stimulation […] And he keeps asking: “what are they doing to help me get better?” so he thinks that the physiotherapy is part of the hospital. He doesn’t know that we’re paying separately. He thinks he’s there to get better. Not to just die.” (BC, Caregiver#2)*

The participants also discussed some positive experiences with the delivery of care, notably the presence of geriatric programs in some acute care settings, and the availability of home care services:

*“My father was able to stay home for the last five years, because we have had support from our system. Until last March, he was at home and had, five times a week, twice a day, people who came to help him at home. If this support had not been there, it would not have been possible for him to stay home” (QC, Caregiver#4)*

### Social environment

Participants discussed how social isolation might increase the risks of later development of frailty. As stated by an HCP:

*“Lonely patients are more vulnerable and have a greater demand for acute care services.” (AB, HCP#2)*

Participants emphasized that HCP and the system must become ‘vigilant’ to identify and support patients at risk of social isolation, and prevent it by increasing public awareness, strengthening ties to the community, and improving relational continuity between family physicians and caregivers (Tables 6 and 7).

While family and friends represent significant sources of social support for frail seniors, caregiver burden remains unaddressed in our healthcare systems:

*“The family caregivers are the shadow work force. […] we recognize they are being burned out. They are being stressed out (…). We are realizing that a lot of the caregiving will fall onto one person in the family. […] when it’s all the spouses looking after the older adult, looking after an older adult can turn into a full-time job.” (AB, HCP#4)*

Participants emphasized that care for frail seniors cannot be effectively improved unless caregiver supports are improved.

*“I think there is too much reliance on public agencies for something as personal as your own care. I think care should be built around family resources, wherever possible. Any resources should be in terms of support to the caregivers, whether they are financial, from the public agencies, or otherwise. The family lies at the center of the healthcare model that I see, with support from outside, whether it’s private or public, tax, or services from the government.”(BC, frail senior#1)*

Moreover, with an aging immigrant population, there is a greater need than before for cultural cohesiveness to address language barriers, cultural beliefs, and expectations of family members and caregivers, especially in end-of-life care.

*“It can be a cultural norm for a family to rally around and take care of their elderly, even if they need a lot of care at home. The staff might then think that it is too unsafe to discharge a patient home. But the family may be adamant that this is what they want to do, even if they know there’s a risk taking them home. But the care team is still hesitant because they want what’s best physically for the patient. Some social sensitivities don’t get the attention they deserve.” (AB, DM#3)*

### Healthcare systems

*Models of Healthcare Delivery.*

Overall, participants shared that the current organization and delivery of healthcare services are system-driven and inappropriate for frail patients:

*“[…] our responses tend to be system driven, and not necessarily patient or family driven. And that’s where we can have problems and gaps in the care that people receive for quality of life.” (BC, HCP#2)*

According to the participants, the division between incurable and curable conditions is inappropriate in the context of frailty. There is a need for holistic, person-centred care that caters to the needs of patients:

*“A person-centred approach helps identify what is important and meaningful for them [frail patients]. It is an interdisciplinary approach where you are including the client and the family, the family physicians, and any other consulting physician, as well as the nurse… and other team members who are needed to support the individual successfully.” (AB, DM#2)*

Improvements in the delivery of healthcare for frail patients should focus on integration of medical and social care, and on improving primary care while facilitating access to other levels of care. Participants identified several promising models suitable for frail patients, notably those to prevent delirium by creation of senior-friendly acute care settings, and the model aiming to improve care integration by delivering primary care services in geriatric clinics. One participant also discussed how the palliative approach fits frail seniors:

*“[…] If you have a terminal diagnosis and you want to stay at home, palliative care is very good and very comprehensive. They have teams of doctors, a partnership with home care, coordinators who are knowledgeable about palliative care, and RNs or nurse practitioners who also have expertise in palliative care. For frailty, it isn’t the same network of teams, but it’s robust. So I think that would be a model to which to aspire to.” (ON, HCP#2)*

#### Cost of care

Participants perceived that while the cost of frail seniors care is high, it still does not meet the needs of frail seniors. The participating HCP mentioned that the funding programs supporting this population are limited, and that fee-for-service limits the delivery of proper care. Services that are deemed useful to frail patients are largely inaccessible and unaffordable to them. Although home care has been successful in supporting frail patients, it is simply insufficient. Thus, new funding models should consider frailty and support of informal caregivers:

*“There needs to be some recognition for the role, whether it’s a tax system approach where you get a reimbursement and have a paid leave when you support someone to the end of life, […] or rest services, which could include things like day programs, or home care support. That type of thing would enable people who are supporting someone who’s frail to have a break.” (AB, DM#2)*

#### Continuity of care

Care transitions can seriously challenge relational continuity and the therapeutic relationship between a patient and the HCP. Participants reported communication breakdowns occurring during care transitions (between different facilities and between floors of the same facility) which can result in serious aggravations for frail patients:

*“Just last week, they sent a patient who could not move out of his chair, home. The note said ‘discharge to long-term care’, but they did not understand... and so, he went home and had a fall. This is within 24 hours of being sent home. So he ended up back in hospital. It’s really about communication. It’s not about medical care.” (ON, HCP#2)*

Transfers from home to residential settings were described as particularly problematic because they could precipitate the onset of mental health issues and rapid decline. The involvement of family physicians or social workers was viewed as essential to prevent worsening and ensure continuity of care. Overall, the predominant perception was that care transitions are not designed nor coordinated to meet the needs of frail patients:

*“We need a sort of a one-stop approach to support frail people when they need to go for a diagnostic testing like blood tests and X-rays, anything like that. Right now, if there’s any kind of investigation that happens… there are probably three or four or five appointments that they have to make on different days, and that just increases the complexity of navigating and getting to an appointment.” (AB, DM#1)*

#### Coordination of care

Participants perceived an important fragmentation in care and services:

*“…having access to appropriate support in home… and the coordination of those services if they are available with healthcare planning and service delivery of primary care... The coordination or all of that is broken, it’s just... it’s embarrassing to explain to my patients... I can’t help them.” (BC, HCP#1)*

Referrals are generally not built into the process of care. Participants underlined how the lack of communication among providers is a recurring issue across the continuum of care.

*“[Note from the author: the participant is discussing the case of an older patient discharged home after being hospitalized for a vertebral fracture] I was unable to contact the people who had discharged her. I called the local community services center, left messages twice, and I was unable to speak to a person to ask questions and have a minimum of information, namely: Was someone in charge of this file? What services had been planned? This, for me, is an important gap. It takes a mechanism to quickly transmit information that a patient has been hospitalized, what she is suffering from, that we are preparing to discharge her, the services that we intend to put in place, the professional to be contacted. The other thing is the connection between family physicians and home care. Even when calling a hot line at the local community services center, access is not easy. These people are having trouble joining us and we are having trouble getting in touch with them. We have to think more about those processes. Effective modes of communication should be established.” (QC, HeCP#4)*

Suggestions for improvement included the use of appropriate communication technology, redesign of care pathways and promotion of patient navigator roles, as discussed by this healthcare provider from British Columbia:

*“Sometimes it’s very confusing to patients and their families who is in charge, who’s involved, who should they be talking to. Maybe a case manager or someone could guide patients and give them more support. Right now, case managers in the community have huge caseloads, so that something might be considered to try to lower the caseloads so that patients and families have a person to turn to give them more time helping them, find the right resources and find the right supports and navigate the system.” (BC, HCP #2)*

### Healthcare organizations

#### Staff management and professional development

Participants stated that staff management influences the quality of frail patient care. They mentioned issues with having the right staff and sufficient staff to attend to the needs:

*“…geriatricians can put all the comorbidities together and come up with a goal of care and plan for the family, and then a plan for the future. I think the family physician is very busy... I don’t think they have time to do it and maybe not the skill set and the expertise at that point. So, I think we need to hire lots of geriatricians.” (AB, HCP#2)*

Participants recommended getting staff more downtimes to prevent burnouts. Other suggestions for improvement focused on the recruitment of geriatricians in primary care and residential facilities and allied healthcare workers to assist with activities of daily living.

*“And I think we need more care workers who are interested in providing care for elders. Not just doctors, but allied health as well.” (ON, HCP#1)*

Many participants felt that HCP lacked training to care for frail patients, yet training was not uniformly available at every healthcare organization:

*“The front line staff has no training (…) yet they may be the clinical assistants, they may be sitting with the patient, they may be caring for the patient, but they have zero education… I think all medical students should go through geriatrics as part of their education. Definitely. […] Nurses too. Everybody.” (ON, HCP#4)*

Participants recommended that training curriculum include topics such as care for dementia, end of life, and guidance for appropriate level of care. They underline the importance for primary care providers to become more aware of supports for frail patients in the community and to improve their skills to identify and care for frail patients.

*“I think all family physicians will be seeing more and more frail elders. So we need to create some capacity for people to take on those patients, both at home and in ambulatory setting, and to feel more competent to take care of them and to be knowledgeable about resources available. I think many family doctors don’t know a lot of resources other than the hospital and the office. So, we need to make them aware of rehabilitation facilities or day treatment programs, respite programs, and all those resources I think aren’t on the radar of most family doctors.” (ON, HCP#2).*

#### Design of Healthcare Facilities and Material Resources

There is a need for senior-friendly environments in all facilities and organizations caring for frail patients:

*“We need to be more aware of senior-friendly environments that will support older adults (…): appropriate signage, appropriate ways of speaking with people who might be hard of hearing, environments that are unthreatening and low in stimulations that exacerbate cognitive behaviours.” (AB, DM#1)*

Overall, there is also a lack of resources (beds, resources for bathing and daily activities) in patient homes and residential facilities.

### Comparisons between types of participants

Participants of any type converged in their interest for care processes, which they all discussed extensively. However, some themes were mentioned exclusively by the DM and HCP, including models of care, education and training of HCP, healthcare staff management, and information sharing. On the other hand, patients and caregivers criticized several aspects of care delivery that were not discussed by HCP and DM, such as bereavement care in hospital settings, the invasion of their intimacy by HCP at home, and the lack of adaptation of long-term care facilities to senior needs. They also brought their unique perspective on assessment, by criticizing the fact that diagnoses are not integrated. Regarding relocalization, they were the only ones among all types of participants to discuss the pressure felt by FS in selecting a residential facility, the failure of transfer plans in meeting FS needs, and the difficult adjustment experienced by FS after transfer. They also mentioned the lack of information sharing between caregivers and patient.

### Comparisons between provinces

Several of the weaknesses and strengths of the current healthcare services were discussed by participants from every province (Tables 6 and 7). On the other hand, several of these themes were raised by participants from multiple provinces, but not from every province, including: information sharing, patient engagement in decision-making, care planning, continuity of care, and material resources and environmental design of healthcare facilities. Within the social environment theme, a single decision maker from Alberta discussed culture, whereas participants from every province discussed social support and social isolation.

Regarding areas of improvement of care processes, the participants from Alberta discussed all sub-themes, but primarily the delivery of care, and care planning and assessment (Additional file 1: Appendix 2). In British Columbia, more participants discussed how to improve the delivery of care and patient involvement in decision-making compared to other sub-themes. The main area of improvement discussed by Nova Scotia participants was delivery of care. Three of the five participants from Ontario made suggestions to improve care processes, specifically assessment, delivery of care, care planning and patient involvement in decision-making. Major areas of improvement discussed in Quebec were assessment, information-sharing and patient engagement in decision-making.

Participants from every province discussed potential improvements to the current social environment, models of delivery of care, and coordination of care. On the other hand, participants from multiple provinces, but not all of the provinces, discussed potential improvements of the other features of the quality of care and services for frail seniors.

## Discussion

We described the perspectives of frail seniors, caregivers, healthcare providers and decision makers from five Canadian provinces on the current state of healthcare and social services for frail seniors. The study participants highlighted needs and care trajectories of frail seniors which are different from those of the general population, and for which the current system is inappropriate. Interpretation of our findings suggest redesigning five main aspects of care and services to meet the specific needs of frail seniors better: access, assessment, communication with frail people and their caregivers, targeted care and services, and integrated care.

Participants highlighted that frail seniors need better access to preventative care and services, and to primary care, in order to avoid functional decline. A scoping literature review concluded that access to community-based primary healthcare can be improved for vulnerable populations by a formal integration of services, while also reducing hospitalizations, emergency department admissions, and unmet healthcare needs [15]. Another recent review concluded that integrated care improved access to care for older people, and quality of care [16].

Study participants also discussed assessment, which they found inadequate for frail seniors. They proposed broader implementation of comprehensive geriatric assessment, and an earlier assessment of geriatric syndromes and frailty in primary care. Hence, a redesign of the healthcare and services systems to meet their specific needs would firstly identify earlier those who are frail. Comprehensive geriatric assessment is the evidence-based process to detect and grade frailty, but is resource-intensive [17]. Ongoing research is trying to improve frailty identification and integrate it into clinical practice for older surgical patients [18], older adults in the emergency department [19] and community-dwelling seniors [20].

Study participants also pointed to specific weaknesses in communication and decision-making processes in the care of frail seniors. They highlighted inadequate sharing of information among patients, caregivers, and healthcare providers, and the lack of involvement of frail seniors and their caregivers in decision-making. These findings are consistent with other studies, which concluded that the routine identification of frailty should trigger important discussions between the interprofessional team, caregivers, and the frail seniors to identify their goals of care and preferences, and choose the care plan that best meet these goals [21]. Despite being less used to an active role in decision-making compared to younger patients there are several examples that, with encouragement, older adults can participate in shared decision-making [22, 23]. Older patients’ active engagement in their healthcare is associated with high-quality and cost-effective healthcare [24, 25]. Identification of frailty, and engagement of seniors in decision-making can in turn offer opportunities for targeted care and services meeting their specific needs [26]. For example, physical and occupational therapy should be offered to improve strength and functioning, and enhance home-based care to help older adults who wish to remain at home [27]. Our findings, however, also suggest that more personalized care requires strengthening HCP capacity to care for seniors with frailty. Study participants indeed discussed the lack of training of healthcare providers to care for frail patients, especially those living with cognitive impairment. HCP training might be challenging as residents and newly graduated family physicians report little interest in caring for seniors afflicted with complex or chronic diseases, especially in home care settings [28]. New training models, such as clinical placements in long-term care settings [29], and interprofessional faculty development programs in geriatrics [30, 31] show promise to improve the autonomy of HCP in their clinical assessment and decision-making with vulnerable older adults.

We also found that the key stakeholders whom we interviewed perceived a need for a more comprehensive, integrated, and interprofessional approach in caring for frail seniors. This suggests that the various healthcare and social services should work together better to address the complex needs of frail patients and their caregivers. Experts in the care of frail seniors suggest that care of the frail seniors should be integrated by balancing medical and non-medical factors, such as nutrition, living situation, function, severity of symptoms, survival and other patient-reported outcomes measures [27]. Interpretation of our findings suggest that achieving this balance requires coordination of a variety of services beyond healthcare, specialized training of providers and families, caregiver support, and information technology and protocols that facilitate effective communication among healthcare providers, patients and their caregivers. Integration of healthcare and social services for frail patients with complex needs has been attempted successfully in health systems around the world [32]. These models were successful in reducing caregiver burden, rates of hospital admission, and delays in care transfer, while limiting overall costs [32]. Integrated funding for healthcare and social services can also potentially improve frail seniors’ access to care, coordination of care, quality of care and health outcomes, while limiting costs of care [33].

### Limitations

Because we used a qualitative approach, our findings are not generalizable to all frail patients, their caregivers, providers and decision makers. The sample also comprised a limited proportion of patients and caregivers among the study participants (8/42), all of whom from QC and BC. Recruiting frail seniors using posters in geriatric clinics proved inefficient in the current study. This might be ascribed to difficulties reading the poster due to vision problems, lack of understanding, or poor health [34]. Recruitment of members of this population through their HCPs, although more efficient, did not either allow recruiting enough participants to ensure saturation within this population. This report might thus not fully represent the perspectives of patients and caregivers, which were often complementary to the perspectives of other types of participants. We, however, gathered the perspectives of stakeholders from five provinces, thus ensuring transferability of our findings to diverse areas of Canada. The majority of our participants were women; however, our findings relate to issues that are similarly experienced by men. We used a convenience sample, so the participants who accepted to participate may be different from the general population.

## Conclusions

Study participants discussed more the weaknesses than the strengths of the current healthcare and social services available to frail seniors. Overall, our findings suggest that frail people require comprehensive assessments, care in continuous relation with various care and service providers, and away from acute care settings. Our findings also stress the importance of integrating care across care settings and over time, of supporting the engagement of patients and caregivers in decision-making so that care and services are adapted to the specific needs and priorities of frail seniors, and of training our workforce to adopt practices that meet the needs of this vulnerable population. Our findings could help redesign healthcare systems more sensitive to the needs and values of frail seniors.

## Additional file

Additional file 1: Detailed themes and subthemes from the qualitative data analyses. Tables (Appendices 1–15) with the count of themes and sub-themes, and for each province. (DOCX 70 kb)

## Abbreviations

AB: Alberta
BC: British Columbia
DM: Decision makers from nursing homes, hospices, hospitals, and home care agencies
HCP: Healthcare provider
NS: Nova Scotia
ON: Ontario
QC: Quebec
