# Supplementary material for: Key stakeholders’ views on the quality of care and services available to frail seniors in Canada
Source: BMC Geriatr. 2018 Nov 26;18:290. doi: 10.1186/s12877-018-0969-y (PMC6260583; doi:10.1186/s12877-018-0969-y)
Supplement: Supplementary file 1 — Detailed themes and subthemes from the qualitative data analyses. Tables (Appendices 1–15) with the count of themes and sub-themes, and for each province. (DOCX 70 kb) [file 12877_2018_969_MOESM1_ESM.docx]

# Appendix 1. Quality of the current healthcare for frail seniors (FS) in Canada, at level of the healthcare processes, notably (1) access (2) assessment (3) information sharing (4) patient involvement in decision making (5) care planning (6) care delivery,

## 1. Frequency of participants who discussed the quality of access to healthcare.

|  | Overall*  (n=22) | Province | | | | |
| --- | --- | --- | --- | --- | --- | --- |
|  |  | AB  (n=3) | BC  (n=7) | NS  (n=3) | ON  (n=3) | QC  (n=6) |
| **Weaknesses** | 19 | 3 | 5 | 3 | 3 | 5 |
| WAITING TIME | 9 | 1 | 1 | 1 | 1 | 5 |
| *Home care programs or services in community* | 3 | 0 | 0 | 0 | 1 | 2 |
| *Acute care settings* | 3 | 0 | 1 | 0 | 0 | 2 |
| *Patients wait too long and end up using ED* | 2 | 1 | 0 | 1 | 0 | 0 |
| CRITERIA OF ACCESS | 8 | 2 | 2 | 1 | 1 | 2 |
| *FS is not meeting specific criteria* | 6 | 2 | 2 | 0 | 1 | 1 |
| ACCESS TO A FAMILY PHYSICIAN | 3 | 1 | 1 | 1 | 0 | 0 |
| *Increase in retired physicians and refusal to accept more patients restrict access to care* | *2* | *1* | *1* | *0* | *0* | *0* |
| TRANSPORT | 3 | 1 | 0 | 1 | 1 | 0 |
| *Travelling to one or more appointment is difficult for patients* | 2 | 1 | 0 | 1 | 0 | 0 |
| **Strengths** | 3 | 0 | 2 | 0 | 0 | 1 |

*Results with an overall n<2 are not shown.

## 2. Frequency of participants who discussed the quality of assessment

|  | Overall*  (n=23) | Province | | | | |
| --- | --- | --- | --- | --- | --- | --- |
|  |  | AB  (n=7) | BC  (n=5) | NS  (n=2) | ON  (n=2) | QC  (n=7) |
| **Weaknesses** | 20 | 7 | 5 | 2 | 1 | 5 |
| INADEQUATE ASSESSMENT | 13 | 6 | 1 | 2 | 0 | 4 |
| MENTAL HEALTH ASSESSMENT | 4 | 3 | 1 | 0 | 0 | 0 |
| TIME CONSTRAINTS DO NOT FAVOUR FS ASSESSMENT | 3 | 3 | 0 | 0 | 0 | 0 |
| LACK OF IDENTIFICATION OF SENIORS WHO ARE FRAIL | 2 | 0 | 1 | 0 | 0 | 1 |
| SOME FS DO NOT HAVE THEIR NEEDS MET | 2 | 1 | 0 | 0 | 0 | 1 |
| **Strengths** | 3 | 0 | 0 | 0 | 1 | 2 |

*Results with an overall n<2 are not shown.

## 3. Frequency of participants who discussed the quality of information sharing

|  | Overall*  (n=8) | Province | | | | |
| --- | --- | --- | --- | --- | --- | --- |
|  |  | AB  (n=2) | BC  (n=3) | NS  (n=2) | ON  (n=0) | QC  (n=1) |
| **Weaknesses** | 8 | 2 | 3 | 2 | 0 | 1 |
| INFORMATION SHARING PROBLEMS WITH CAREGIVERS | 4 | 1 | 1 | 1 | 0 | 1 |
| INFORMATION SHARING PROBLEMS BETWEEN FS WITH COGNITIVE IMPAIRMENTS AND HCP | 3 | 1 | 1 | 1 | 0 | 0 |
| LACK OF EVIDENCE REGARDING THE EFFICACY OF TREATMENTS BEING AN OBSTACLE TO DISCUSSIONS | 1 | 0 | 1 | 0 | 0 | 0 |

*Results with an overall n<2 are not shown.

## 4. Frequency of participants who discussed the quality of patient engagement in decision-making

|  | Overall*  (n=11) | Province | | | | |
| --- | --- | --- | --- | --- | --- | --- |
|  |  | AB  (n=2) | BC  (n=5) | NS  (n=0) | ON  (n=2) | QC  (n=2) |
| **Weaknesses** | 9 | 2 | 4 | 0 | 1 | 2 |
| LACK OF INCLUSION OF PATIENT IN THE DECISION-MAKING PROCESS | 6 | 0 | 3 | 0 | 1 | 2 |
| FAMILY’S INVOLVEMENT | 2 | 2 | 0 | 0 | 0 | 0 |
| *Family decision could be in disagreement with HCP perspective on quality* | 2 | 2 | 0 | 0 | 0 | 0 |
| **Strengths** | 4 | 0 | 2 | 0 | 1 | 1 |
| ADVOCACY FOR THEMSELVES - PATIENTS | 3 | 0 | 2 | 0 | 1 | 0 |

*Results with an overall n<2 are not shown.

## 5. Frequency of participants who discussed the quality of care planning.

|  | Overall*  (n=4) | Province | | | | |
| --- | --- | --- | --- | --- | --- | --- |
|  |  | AB  (n=1) | BC  (n=0) | NS  (n=1) | ON  (n=0) | QC  (n=2) |
| **Weaknesses** | 3 | 1 | 0 | 1 | 0 | 1 |

*Results with an overall n<2 are not shown.

## 6. Frequency of participants who discussed the quality of care delivery

|  | Overall*  (n=30) | Province | | | | |
| --- | --- | --- | --- | --- | --- | --- |
|  |  | AB  (n=6) | BC  (n=10) | NS  (n=4) | ON  (n=2) | QC  (n=8) |
| **Weaknesses** | 29 | 6 | 10 | 4 | 2 | 7 |
| SETTING | 17 | 1 | 8 | 2 | 1 | 5 |
| *Hospital and acute care settings* | 8 | 1 | 3 | 0 | 0 | 4 |
| Inadequate interventions | 6 | 1 | 2 | 0 | 0 | 3 |
| *Home* | 7 | 0 | 3 | 2 | 0 | 2 |
| Lack or insufficient home support (resources or services from community or healthcare network ) | 6 |  | 3 | 2 |  | 1 |
| *Residential facilities* | 5 | 0 | 2 | 0 | 1 | 2 |
| Lower quality of life | 2 | 0 | 1 | 0 | 0 | 1 |
| Lack or insufficient service | 2 | 0 | 1 | 0 | 1 | 0 |
| STIGMA AND PREJUDICES AGAINST FS | 9 | 5 | 0 | 1 | 0 | 3 |
| MEDICATION | 7 | 1 | 2 | 2 | 1 | 1 |
| *Inadequate use of medication to FS (either HCP or patient)* | 4 | 0 | 0 | 2 | 1 | 1 |
| *Unattended consequences of medication (risks with FS)* | 3 | 1 | 2 | 0 | 0 | 0 |
| MENTAL HEALTH ISSUE | 6 | 0 | 3 | 1 | 1 | 1 |
| *Inadequate response to agitated patients (managing responsive behaviours)* | 3 | 0 | 2 | 0 | 0 | 1 |
| **Strengths** | 8 | 1 | 2 | 0 | 1 | 4 |
| AVAILABILITY OF SERVICES AND HOME CARE PROGRAMS | 4 | 0 | 0 | 0 | 1 | 3 |

*Results with an overall n<2 are not shown.

# Appendix 2. Frequency of participants who discussed potential improvements to the current care processes for FS in Canada

|  | Overall*  (n=30) | Province | | | | |
| --- | --- | --- | --- | --- | --- | --- |
|  |  | AB  (n=7) | BC  (n=11) | NS  (n=3) | ON  (n=3) | QC  (n=6) |
| **1.Access to healthcare** | 12 | 4 | 0 | 2 | 2 | 4 |
| MORE ACCESS TO CARE IN THE COMMUNITY | 3 | 2 | 0 | 0 | 0 | 1 |
| MORE ACCESS TO SPECIALITY CARE | 3 | 2 | 0 | 0 | 0 | 1 |
| HOSPITAL OR ED AVOIDANCE AS MUCH AS POSSIBLE | 2 | 1 | 0 | 0 | 1 | 0 |
| HAVING TRANSPORT AND LOGISTIC TO HEALTH SERVICES | 2 | 1 | 0 | 0 | 0 | 1 |
| **2. Assessment** | 13 | 4 | 2 | 0 | 2 | 5 |
| UNDERSTANDING THE NEEDS OF FS | 8 | 2 | 1 | 0 | 0 | 5 |
| SPECIFIC DIAGNOSTIC TECHNIQUES AND PROCEDURES | 4 | 3 | 0 | 0 | 1 | 0 |
| IMPROVED DIAGNOSIS OF FRAILTY AMONGST THE ELDERLY | 3 | 1 | 1 | 0 | 0 | 1 |
| **3. Information sharing** | 3 | 1 | 1 | 1 | 0 | 0 |
| HAVING A PROTOCOL FOR TRANSPARENT DISCUSSIONS | 2 | 0 | 1 | 1 | 0 | 0 |
| **4. Patient engagement in decision-making** | 9 | 1 | 3 | 1 | 1 | 3 |
| OFFERING FS OPTIONS TO DECIDE WHERE TO LIVE | 4 | 0 | 1 | 1 | 1 | 1 |
| INVOLVING FS AND CAREGIVERS IN THE DECISION-MAKING PROCESS | 3 | 0 | 1 | 0 | 0 | 2 |
| PROVIDING THE RESOURCES TO SUPPORT THEIR DECISIONS | 2 | 1 | 1 | 0 | 0 | 0 |
| **5. Care planning** | 7 | 4 | 0 | 1 | 1 | 1 |
| CREATING INTEGRATED CARE PLANS | 5 | 4 | 0 | 0 | 0 | 1 |
| **6. Care delivery** | 19 | 5 | 7 | 3 | 1 | 3 |
| COMMUNITY AND HOME | 16 | 5 | 6 | 2 | 1 | 2 |
| *More activities and care at home* | 10 | 3 | 4 | 1 | 0 | 2 |
| *More activities, resources and care in the community* | 10 | 4 | 3 | 1 | 1 | 1 |
| RESIDENTIAL FACILITIES | 2 | 0 | 1 | 0 | 0 | 1 |

*Results with an overall n<2 are not shown.

# Appendix 3. Quality of the current healthcare for frail seniors (FS) in Canada, at level of the social environment.

|  | Overall*  (n=23) | Province | | | | |
| --- | --- | --- | --- | --- | --- | --- |
|  |  | AB  (n=6) | BC  (n=6) | NS  (n=3) | ON  (n=3) | QC  (n=5) |
| **Social support** | 19 | 5 | 5 | 2 | 3 | 4 |
| **Weaknesses** | 16 | 5 | 4 | 2 | 3 | 2 |
| BURDEN OF CAREGIVERS | 9 | 2 | 2 | 1 | 2 | 2 |
| *Caregivers feel stressed or exhausted* | 4 | 1 | 1 | 1 | 0 | 1 |
| *Caregivers feel compelled to help FS in health facilities* | 2 | 0 | 2 | 0 | 0 | 0 |
| *Some caregivers do not know how to tolerate the complexity of care of FS* | 2 | 1 | 0 | 1 | 0 | 0 |
| Challenges for caregivers to support FS with dementia | 2 | 0 | 0 | 0 | 1 | 1 |
| LACK OF FAMILY SUPPORT | 7 | 3 | 3 | 1 | 0 | 0 |
| *FS without family support could have more problems to receive care and services* | 6 | 2 | 3 | 1 | 0 | 0 |
| PROBLEMS WITH PATIENT ADVOCACY | 2 | 0 | 1 | 0 | 1 | 0 |
| **Strengths** | 9 | 1 | 3 | 1 | 1 | 3 |
| SUPPORT FROM CAREGIVERS, FAMILY MEMBERS AND COMMUNITY | 6 | 1 | 1 | 1 | 0 | 3 |
| ADVOCACY BY CAREGIVERS OR FAMILY MEMBERS ALLOWED BETTER CARE OR RESOURCES | 3 | 0 | 2 | 0 | 1 | 0 |
|  |  |  |  |  |  |  |
| **Social isolation** | 10 | 2 | 3 | 1 | 2 | 2 |
| **Weaknesses** | 8 | 2 | 2 | 1 | 2 | 1 |
| UNRESOLVED FEELINGS OF LONELINESS | 5 | 1 | 2 | 1 | 1 | 0 |
| LIVING ALONE AT HOME WITHOUT SUPPORT | 3 | 1 | 0 | 0 | 1 | 1 |
| **Strengths** | 4 | 2 | 1 | 0 | 0 | 1 |

*Results with an overall n<2 are not shown.

# Appendix 4. Frequency of participants who discussed potential improvements to the social environment for FS in Canada

|  | Overall*  (n=13) | Province | | | | |
| --- | --- | --- | --- | --- | --- | --- |
|  |  | AB  (n=3) | BC  (n=5) | NS  (n=1) | ON  (n=1) | QC  (n=3) |
| **Social support** | 12 | 3 | 5 | 1 | 0 | 3 |
| IMPROVING SUPPORT GIVEN TO CAREGIVERS AND FAMILY MEMBERS | 10 | 3 | 3 | 1 | 0 | 3 |
| HAVING SUPPORT FROM CAREGIVERS, FAMILY MEMBERS OR THE COMMUNITY | 4 | 2 | 2 | 0 | 0 | 0 |
| **Social isolation** | 2 | 0 | 1 | 0 | 1 | 0 |

*Results with an overall n<2 are not shown.

# Appendix 5. Quality of the current healthcare for frail seniors (FS) in Canada, at level of models of delivery of care.

|  | Overall*  (n=20) | Province | | | | |
| --- | --- | --- | --- | --- | --- | --- |
|  |  | AB  (n=7) | BC  (n=4) | NS  (n=3) | ON  (n=2) | QC  (n=4) |
| **Weaknesses** | 18 | 6 | 4 | 3 | 1 | 4 |
| HOLISTIC APPROACH | 12 | 4 | 3 | 2 | 1 | 2 |
| *Lack of a holistic approach* | 11 | 4 | 3 | 2 | 1 | 1 |
| *It remains a challenge to promote a holistic approach* | 2 | 1 | 0 | 0 | 0 | 1 |
| ACTUAL MODEL OF CARE IS INAPPROPRIATE | 5 | 1 | 1 | 1 | 0 | 2 |
| *Care is system-driven, not necessarily patient or family-driven* | 3 | 1 | 1 | 0 | 0 | 1 |
| *The current model - incurable vs curable illness - is inadequate* | 2 | 0 | 0 | 1 | 0 | 1 |
| FRAILTY CONCEPT UNCOMMON OR INADEQUATE | 2 | 1 | 0 | 1 | 0 | 0 |
| **Strengths** | 4 | 2 | 1 | 0 | 1 | 0 |
| PRIMARY CARE DELIVERY IN GERIATRIC CLINICS TO IMPROVE CARE INTEGRATION | 2 | 2 | 0 | 0 | 0 | 0 |

*Results with an overall n<2 are not shown.

# Appendix 6. Frequency of participants who discussed potential improvements to the current models of delivery of care for FS in Canada.

|  | Overall*  (n=25) | Province | | | | |
| --- | --- | --- | --- | --- | --- | --- |
|  |  | AB  (n=8) | BC  (n=4) | NS  (n=3) | ON  (n=4) | QC  (n=6) |
| NECESSITY OF DELIVERING PATIENT-CENTRED CARE | 21 | 7 | 2 | 3 | 3 | 6 |
| *To promote a person-centered approach through collaboration between HCP* | 11 | 5 | 2 | 2 | 1 | 1 |
| *More holistic approach of the patient need* | 7 | 3 | 1 | 2 | 1 | 0 |
| *Promoting healthcare adapted to patient's need* | 4 | 0 | 0 | 0 | 1 | 3 |
| *Programs, approaches and environments adapted to FS with mental health issues* | 3 | 0 | 1 | 0 | 1 | 1 |
| *FS must be the basis of a new model of care and health system* | 3 | 1 | 0 | 0 | 0 | 2 |
| BETTER INTEGRATION OF CARE | 5 | 2 | 1 | 1 | 0 | 1 |
| IMPROVE PRIMARY CARE AND ACCESS TO OTHER LEVELS OF CARE | 4 | 4 | 0 | 0 | 0 | 0 |

*Results with an overall n<2 are not shown.

# Appendix 7. Quality of the current healthcare for frail seniors (FS) in Canada, at level of the cost of care

|  | Overall* (n=17) | Province | | | | |
| --- | --- | --- | --- | --- | --- | --- |
|  |  | AB  (n=4) | BC  (n=3) | NS  (n=2) | ON  (n=2) | QC  (n=6) |
| **Weaknesses** | 17 | 4 | 3 | 2 | 2 | 6 |
| HIGH COSTS FOR HEALTHCARE NETWORK | 7 | 2 | 0 | 1 | 0 | 4 |
| *Providing care to FS is expensive to the health network* | 6 | 2 | 0 | 1 | 0 | 3 |
| *A poor targeting of patients’ needs is expensive for the health network* | 3 | 1 | 0 | 0 | 0 | 2 |
| HEALTH AND SERVICES EXPENSES INCURRED BY PATIENTS AND FAMILY MEMBERS | 6 | 0 | 3 | 0 | 1 | 2 |
| *Living in private residential facilities* | 2 | 0 | 0 | 0 | 0 | 2 |
| *Medication* | 2 | 0 | 2 | 0 | 0 | 0 |
| *Physiotherapy services* | 2 | 0 | 2 | 0 | 0 | 0 |
| *Travel and parking expenses* | 2 | 0 | 2 | 0 | 0 | 0 |
| THE MODEL OF HCP REMUNERATION LIMITS THE ASSESSMENT OF FS | 5 | 2 | 0 | 1 | 1 | 1 |
| FUNDING | 3 | 2 | 0 | 1 | 0 | 0 |
| *Lack of funding for FS programs* | 3 | 2 | 0 | 1 | 0 | 0 |

*Results with an overall n<2 are not shown.

# Appendix 8. Frequency of participants who discussed potential improvements to the cost of care of FS in Canada.

|  | Overall*  (n=6) | Province | | | | | |
| --- | --- | --- | --- | --- | --- | --- | --- |
|  |  | AB  (n=2) | BC  (n=1) | NS  (n=1) | ON  (n=0) | QC  (n=2) |  |
| FUNDING TO SUPPORT CAREGIVER AND FAMILY | 3 | 1 | 1 | 0 | 0 | 1 |  |
| PHYSICIAN FUNDING MODELS ADAPTED TO FS CARE MUST BE DEVELOPED AND PROMOTED | 2 | 1 | 0 | 1 | 0 | 0 |  |

*Results with an overall n<2 are not shown.

# Appendix 9. Quality of the current healthcare for frail seniors (FS) in Canada, at level of continuity of care, notably (A) relational continuity (B) informational continuity, and (C) management continuity.

## A. Frequency of participants who discussed the quality of relational continuity.

|  | Overall*  (n=10) | Province | | | | |
| --- | --- | --- | --- | --- | --- | --- |
|  |  | AB  (n=0) | BC  (n=2) | NS  (n=0) | ON  (n=1) | QC  (n=7) |
| **Weaknesses** | 10 | 0 | 2 | 0 | 1 | 7 |
| ISSUES WITH PATIENT TRANSFERS | 7 | 0 | 2 | 0 | 0 | 5 |
| *Patient transfers can induce cognitive problems* | 3 | 0 | 1 | 0 | 0 | 2 |
| *Patient transfers from home to a residential facility can be a difficult adjustment* | 2 | 0 | 0 | 0 | 0 | 2 |
| INADEQUATE ATTENTION GIVEN TO THE PERSONAL HYGIENE OF PATIENTS IN ACUTE CARE SETTINGS | 2 | 0 | 0 | 0 | 0 | 2 |
| **Strengths** | 2 | 0 | 1 | 0 | 0 | 1 |

*Results with an overall n<2 are not shown

## B. Frequency of participants who discussed the quality of informational continuity.

|  | Overall*  (n=6) | Province | | | | |
| --- | --- | --- | --- | --- | --- | --- |
|  |  | AB  (n=1) | BC  (n=3) | NS  (n=0) | ON  (n=1) | QC  (n=1) |
| **Weaknesses** | 6 | 1 | 3 | 0 | 1 | 1 |
| PROBLEMS WITH INFORMATION TRANSFER | 6 | 1 | 3 | 0 | 1 | 1 |
| *Inadequate discharge planning from hospital setting to other settings* | 4 | 0 | 2 | 0 | 1 | 1 |
| *Lack of communication between family physicians and HCP* | 2 | 0 | 1 | 0 | 0 | 1 |

## *Results with an overall n<2 are not shown

## C. Frequency of participants who discussed the quality of management continuity.

|  | Overall*  (n=3) | Province | | | | |
| --- | --- | --- | --- | --- | --- | --- |
|  |  | AB  (n=1) | BC  (n=0) | NS  (n=0) | ON  (n=0) | QC  (n=2) |
| **Strengths** | 2 | 1 | 0 | 0 | 0 | 1 |

*Results with an overall n<2 are not shown.

| Appendix 12. Frequency of participants who discussed potential improvements to the current continuity of FS care in Canada**.** | Overall*  (n=8) | Province | | | | |
| --- | --- | --- | --- | --- | --- | --- |
|  |  | AB  (n=3) | BC  (n=2) | NS  (n=1) | ON  (n=0) | QC  (n=2) |
| RELATIONAL CONTINUITY | 5 | 1 | 2 | 0 | 0 | 2 |
| *Availability of HCP in a timely fashion* | 3 | 1 | 1 | 0 | 0 | 1 |
| INFORMATIONAL CONTINUITY | 2 | 1 | 0 | 1 | 0 | 0 |

*Results with an overall n<2 are not shown.

# Appendix 10. Quality of the current healthcare for frail seniors (FS) in Canada, at level of professional development of HCP.

|  | Overall* (n=13) | Province | | | | |
| --- | --- | --- | --- | --- | --- | --- |
|  |  | AB  (n=4) | BC  (n=3) | NS  (n=1) | ON  (n=2) | QC  (n=3) |
| **Weaknesses** | 12 | 4 | 3 | 0 | 2 | 3 |
| LACK OF KNOWLEDGE AMONG HCP ABOUT HOW TO CARE FOR FS | 5 | 3 | 1 | 0 | 0 | 1 |
| LACK OF KNOWLEDGE ABOUT RESPONDING TO AGITATED PATIENTS (managing response behaviours) | 3 | 1 | 0 | 0 | 1 | 1 |
| LACK OF TRAINING | 2 | 0 | 1 | 0 | 1 | 0 |
| **Strengths** | 4 | 1 | 0 | 1 | 2 | 0 |
| PROMOTION OR IMPLEMENTATION OF TRAINING PROGRAMS IN healthcare FOR THE ELDERLY | 4 | 1 | 0 | 1 | 2 | 0 |

*Results with an overall n<2 are not shown.

# Appendix 11. Frequency of participants who discussed potential improvements in the current professional development of HCP.

|  | Overall*  (n=10) | Province | | | | |
| --- | --- | --- | --- | --- | --- | --- |
|  |  | AB  (n=4) | BC  (n=1) | NS  (n=0) | ON  (n=2) | QC  (n=3) |
| IMPROVING GERIATRIC KNOWLEDGE, SKILLS, COMPETENCY | 8 | 4 | 0 | 0 | 1 | 3 |

*Results with an overall n<2 are not shown.

# Appendix 12. Quality of the current healthcare for frail seniors (FS) in Canada, at level of healthcare staff management.

|  | Overall*  (n=8) | Province | | | | |
| --- | --- | --- | --- | --- | --- | --- |
|  |  | AB  (n=1) | BC  (n=4) | NS  (n=0) | ON  (n=1) | QC  (n=2) |
| **Weaknesses** | 7 | 1 | 4 | 0 | 1 | 1 |
| WORKLOAD | 4 | 0 | 3 | 0 | 0 | 1 |
| *Workload of nurses* | 2 | 0 | 1 | 0 | 0 | 1 |
| *Staff workload and lower attention to patient* | 2 | 0 | 2 | 0 | 0 | 0 |
| LACK OF STAFF | 3 | 0 | 2 | 0 | 1 | 0 |
| *Lack of staff in acute care setting to support patient volume* | 3 | 0 | 2 | 0 | 1 | 0 |
| ROTATING STAFF AND LACK OF CONSISTENCY | 2 | 0 | 1 | 0 | 1 | 0 |
| **Strengths** | 2 | 1 | 0 | 0 | 0 | 1 |
| GERIATRICIANS CAN SUPPORT FAMILY PHYSICIANS AND PRIMARY CARE IN GENERAL | 2 | 1 | 0 | 0 | 0 | 1 |

*Results with an overall n<2 are not shown.

# Appendix 13. Frequency of participants who discussed potential improvements in the current management of healthcare staff for FS in Canada.

|  | Overall*  (n=6) | Province | | | | |
| --- | --- | --- | --- | --- | --- | --- |
|  |  | AB  (n=2) | BC  (n=3) | NS  (n=0) | ON  (n=1) | QC  (n=0) |
| MORE STAFF RECRUITMENT | 3 | 1 | 1 | 0 | 1 | 0 |

*Results with an overall n<2 are not shown.

# Appendix 14. Quality of the current healthcare for frail seniors (FS) in Canada, at levels of material resources and environmental design of healthcare facilities.

|  | Overall*  (n=12) | Province | | | | | |
| --- | --- | --- | --- | --- | --- | --- | --- |
|  |  | AB  (n=5) | BC  (n=5) | NS  (n=0) | ON  (n=1) | QC  (n=1) |  |
| **Weaknesses** | 11 | 4 | 5 | 0 | 1 | 1 |  |
| ENVIRONMENTAL DESIGN NOT SUITABLE- ALL SETTINGS | 9 | 4 | 4 | 0 | 0 | 1 |  |
| *Environmental design is not suitable in hospital and acute care settings* | 6 | 4 | 2 | 0 | 0 | 0 |  |
| *Environment design is not suitable at home* | 2 | 1 | 1 | 0 | 0 | 0 |  |
| LACK OR INSUFFICIENT BEDS - ALL SETTINGS | 3 | 1 | 1 | 0 | 1 | 0 |  |
| OTHER RESOURCES | 2 | 0 | 2 | 0 | 0 | 0 |  |
| **Strengths** | 2 | 1 | 1 | 0 | 0 | 0 |  |

*Results with an overall n<2 are not shown.

# Appendix 15. Frequency of participants who discussed potential improvements of the material resources and environmental design of healthcare facilities for FS in Canada.

|  | Overall*  (n=7) | Province | | | | |
| --- | --- | --- | --- | --- | --- | --- |
|  |  | AB  (n=4) | BC  (n=1) | NS  (n=0) | ON  (n=1) | QC  (n=1) |
| ENVIRONMENTAL DESIGN | 5 | 4 | 0 | 0 | 0 | 1 |
| A better environmental design in healthcare settings better suited for patients | 4 | 4 | 0 | 0 | 0 | 0 |

*Results with an overall n<2 are not shown.
